# Mediterranean Journal of Rheumatology March 2018 Highlights

**DOI:** 10.31138/mjr.29.1.11

**Published:** 2018-03-19

**Authors:** George D. Kitas

**Affiliations:** Research and Development Directorate, Dudley Group NHS Foundation Trust, Russells Hall Hospital, Dudley, West Midlands, UK; Arthritis Research UK Centre for Epidemiology, University of Manchester, Manchester, UK

Welcome to the March 2018 issue of MJR: it includes a number of original papers, reviews and case reports which will hopefully be of great interest to our readers.

It is a great honor for MJR to host the first official report of the Greek Nationwide Rheumatoid Arthritis Cohort. Thomas et al.^4^ present data from over 2400 RA patients, constituting one of the largest epidemiologic studies in the literature with real-life data regarding disease characteristics, treatment patterns and comorbidities of RA patients in Greece. We are sure that this collaborative effort will produce ever more interesting data, which will define current and future clinical practice in Greece and abroad. In the editorial of this issue, Dr Nikas discusses the place of corticosteroids in the modern treatment era of rheumatic diseases, especially in rheumatoid arthritis.^1^ The long-term use of low-dose prednisone in various autoimmune disorders has been revisited, and Dr Nikas critically presents the pros and cons of different treatment approaches.

The role of the Mediterranean diet in the context of different – rheumatic or not - disease subsets has become an extremely popular field over the last years. In the forthcoming Mediterranean Congress of Rheumatology in Genoa, the role of diet in Rheumatic Diseases is the central theme. Addressing this emerging interest, Bodganos et al.^3^ provide an extremely useful clinical overview of the beneficial effects of the Mediterranean diet in patients with hyperuricemia and gout. Please note that a future special issue of the Mediterranean Journal of Rheumatology, guest-edited by Dr. Bogdanos, will be entirely focused in this field and we invite submissions to it.

The classification of vasculitic syndromes on the basis of the type of ANCA (MPO_4_ or PR_3_) positivity has emerged as a parameter in the diagnosis and management of small vessels vasculitis, since serology is thought to associate with clinicopathological aspects, disease activity, possibly relapse, and response to therapy. In a mini review, Dr Csernok summarizes the new concepts in ANCA detection and highlights the importance of using specific PR3- and MPO-ANCA immunoassays in daily clinical practice in order to avoid misdiagnosis.^2^

Ziad S. Al-Rawi et al.^5^ studied 111 RA subjects and 97 controls and found that Red cell distribution width (RDW) is significantly higher in the patient group. Given that RDW, as part of a full blood cell count, is a measured parameter performed by an automated analyzer in routine practice, they suggest that it could be used as an additional easily accessible tool for monitoring and managing RA. Future studies may help validate further and position this test in routine practice.

In this issue, MJR readers can also find a number of exceptional case reports, such as uncommon clinical manifestations of granulomatosis with polyangiitis^8^ and systemic lupus erythematosus presenting as liver dysfunction and chorea^6^ respectively, thyrotoxicosis manifested as acute onset muscle weakness and periodic paralysis^7^ and finally a rare case of antithyroid drug induced syndrome with clinical and serological characteristics indicating vasculitis and systemic lupus.^9^

Last but not least, the first research protocols funded competitively after international peer review by the Greek Rheumatology Society and Professional Association of Rheumatologists for the next two years are presented in this issue. This research work will address important questions related to the pathophysiology of lymphoma in Sjögren’s syndrome^10^ and the effect of novel small molecules – namely apremilast – on the activation and differentiation of regulatory B cells in patients with psoriatic arthritis.^11^
